# Telomere Gene Therapy: Polarizing Therapeutic Goals for Treatment of Various Diseases

**DOI:** 10.3390/cells8050392

**Published:** 2019-04-28

**Authors:** JinWoo Hong, Chae-Ok Yun

**Affiliations:** 1 Department of Bioengineering, College of Engineering, Hanyang University, 222 Wangsimni-ro, Seongdong-gu, Seoul 04763, Korea; jhong803@hanyang.ac.kr; 2 Institute of Nano Science and Technology (INST), Hanyang University, Seoul 04763, Korea

**Keywords:** telomere, telomerase, telomere dysfunction, gene therapy, anti-aging, regenerative medicine, cancer therapy

## Abstract

Modulation of telomerase maintenance by gene therapy must meet two polarizing requirements to achieve different therapeutic outcomes: Anti-aging/regenerative applications require upregulation, while anticancer applications necessitate suppression of various genes integral to telomere maintenance (e.g., telomerase, telomerase RNA components, and shelterin complex). Patients suffering from aging-associated illnesses often exhibit telomere attrition, which promotes chromosomal instability and cellular senescence, thus requiring the transfer of telomere maintenance-related genes to improve patient outcomes. However, reactivation and overexpression of telomerase are observed in 85% of cancer patients; this process is integral to cancer immortality. Thus, telomere-associated genes in the scope of cancer gene therapy must be inactivated or inhibited to induce anticancer effects. These contradicting requirements for achieving different therapeutic outcomes mean that any vector-mediated upregulation of telomere-associated genes must be accompanied by rigorous evaluation of potential oncogenesis. Thus, this review aims to discuss how telomere-associated genes are being targeted or utilized in various gene therapy applications and provides some insight into currently available safety hazard assessments.

## 1. Introduction

The eukaryotic nuclear genome is packaged into discrete linear chromosomes, and this poses a unique biological problem: The ends of linear chromosomes must be distinguished from breaks to avoid unnecessary repair processes that may promote end-to-end fusion of chromosomes [1]. To this end, telomeres protect against end-to-end fusion of chromosomes and maintain the genomic stability of cells during division. However, the ends of linear chromosomes cannot be completely replicated by DNA replication machinery, and this leads to progressive shortening of telomeres with each round of DNA replication. The gradual telomere attrition following numerous cycles of cell proliferation promotes DNA damage responses at the chromosome ends, ultimately resulting in senescence and development of various age-related diseases. Furthermore, telomere loss and uncapping of chromosomes induce progressive tissue degeneration, stem cell depletion, organ failure, and dysfunctional tissue repair [2]. Thus, it is logical that various strategies are actively being investigated to combat telomere attrition to rejuvenate and restore the regenerative capacities of aging cells.

The telomere attrition of linear chromosomes is solved in most eukaryotes by specialized telomeric nucleic acid-protein complexes: Telomeric DNA with a repetitive G-rich sequence composed of 5′-TTAGGG-3′ can be synthesized de novo by a complex composed of a catalytic subunit known as telomerase reverse transcriptase (TERT) and telomerase RNA component (TERC) that serves as a template. Additionally, a shelterin complex, which is a protein complex composed of three core subunits (telomeric repeat-binding factor (TRF)1, TRF2, and protection of telomeres protein (POT)1), is also critical for maintenance of telomere stability, as it enables DNA damage response machinery to distinguish telomeres from sites of genomic DNA damage [3]. Thus, vector-mediated regulation of the above-mentioned factors by gene therapy can be a promising strategy to promote telomere elongation and cell regeneration and to combat age-associated illnesses where telomere attrition is a risk factor.

Despite these potential benefits of telomerase gene therapy, constitutive telomerase activation may lead to oncogenesis, since cancer cells frequently reactivate and upregulate telomerase activity to achieve immortalization [4,5]. Cancer cells with oncogenic mutations to telomerase bypass senescence, thus enabling indefinite and rapid proliferation of these cells for disease progression. Thus, telomerase gene therapy in the scope of cancer treatment necessitates gene suppression rather than gene transfer or activation, as in cases of regenerative or anti-aging applications. The polarity of telomerase gene regulation required to achieve different therapeutic goals means that double-edged telomerase gene therapy must be carefully regulated to assess both the therapeutic benefits and the risks. In this review, we aim to discuss how different telomere gene therapy strategies are being investigated to combat various age-associated illnesses and address safety concerns that arise due to the polarizing therapeutic requirements for achieving diverse therapeutic goals.

## 2. Regenerative and Anti-Aging Applications

Telomere attrition causes a wide range of age-related diseases. To this end, transfer of genes essential for telomere maintenance can be a promising strategy to combat these illnesses and restore the regenerative capacity of aging tissues. The first human use of telomerase gene transfer was reported in 2015, but the test was quite unorthodox, as self-experimentation was performed on the CEO of a company developing experimental adeno-associated virus (AAV) expressing human TERT. Despite the skepticism surrounding the results reported by the company, which have not been published in any peer-reviewed academic journal to date and are only available on the company website (https://bioviva-science.com/blog/new-telomere-length-results-a-2018-update-by-liz-parrish/), there are increasing amounts of experimental evidence from other researchers supporting the transfer of the essential genes of telomere maintenance as a promising strategy to combat aging and restore cellular regenerative function for therapeutic benefits.

### 2.1. Anti-Aging: Improving Longevity

Telomere attrition and the resulting senescence are well-known attributes of aging in mammals, and inhibiting senescence has been shown to improve the health span of aging mice [6,7,8,9]. The restoration of TERC in TERC-deficient transgenic (TG) mice with critically short telomeres has been shown to restore chromosomal stability [10], while others have shown that restoration of telomerase activity reverses tissue degeneration in aged mice [11]. These early studies examining the pathophysiology of TG mice with short telomeres provided strong evidence that telomerase gene therapy may be beneficial in anti-aging applications. In support, the constitutive telomerase expression in cancer-resistant TG mice, which showed 10-fold higher activation of telomerase compared to the wild-type mice, exhibited improvements in the fitness of epithelial barriers, systemic delays in aging, and extension of life span [12].

In 2012, nonintegrative AAV serotype 9 (AAV9) vector expressing murine TERT improved the longevity of both adult (1-year-old) and old (2-year-old) mice [13]. AAV9 was utilized due to its ability to transduce a broad range of tissues effectively through the systemic delivery of the virus; this was evidenced by ectopic expression of murine TERT in a wide range of tissues, including liver, kidney, lungs, heart, brain, and muscle, at 1 month post systemic injection of AAV9. The TERT expression levels were maintained for at least 8 months in these tissues, with the exception of the lungs. AAV9-induced TERT expression was comparable to those achieved by transgenesis in mice [12], showing that strong gene transfer can be achieved postnatally in aging mice by a gene transfer vector. Induction of ectopic TERT expression in aging mice showed lower incidences of age-related osteoporosis and glucose intolerance, while improving neuromuscular function and object recognition, showing that the degenerate physiological conditions that develop with aging can be prevented by telomerase gene therapy. In line with these assessments, the actual lifespan of the mice was significantly extended following TERT gene transfer; a significant increase in median survival as well as a 13% or 20% increase in maximum longevity (for mice treated at 1 or 2 years of age, respectively) was observed in the longest-lived mice of the TERT gene transfer group. Interestingly, the pathological analysis of all mice postmortem revealed that ectopic TERT expression by AAV9 did not increase cancer incidence compared to treatment with the GFP-expressing AAV9 control vector. This is in sharp contrast to the TG mouse models, where constitutive overexpression of TERT in the absence of genetically endowed cancer resistance by germline modification increased the cancer incidence rate [14]. Although these discrepancies in the oncogenic potential of ectopically expressed TERT require more extensive evaluation in the future, there is a telomerase-deficient TG mouse model [11] that supports the findings achieved by the TERT transfer of AAV9. Specifically, a TG mouse with a 4-hydroxytamoxifen (4-OHT)-inducible TERT system possesses dysfunctional and short telomeres. The telomere length can be rescued by the conditional and transient reactivation of telomerase by treatment with 4-OHT, and this has been shown to eliminate degenerative phenotypes without promoting carcinogenesis, suggesting that transient TERT expression may circumvent the potential risk of oncogenesis.

Similar improvements in the longevity of aging mice were achieved by transfer of the *TRF1* gene, which is an essential part of the shelterin complex for telomere replication and protection, using AAV9 [15]. The authors argued that a nonintegrative vector was required to induce transient *TRF1* overexpression in adult mice, as their previous work demonstrated that constitutive *TRF1* overexpression in TG mice led to telomere shortening through telomere cleavage by XPF nuclease [16]. Similar to the AAV9-mediated murine TERT expression occurring in an ectopic manner, the *TRF1* gene was expressed in a number of organs, including the liver, heart, muscle, and brain. Ectopic *TRF1* expression induced by AAV9 did not increase the cancer incidence rate in mice, further supporting the notion that transient expression of core telomere maintenance genes does not necessarily lead to carcinogenesis. Interestingly, induction of *TRF1* expression only improved the longevity of old mice (2 years old at treatment), whereas it failed to improve the lifespan of adult mice (1 year at treatment), suggesting that TRF1 overexpression, unlike TERT, can only increase life expectancy when administered during a shorter therapeutic window. Additionally, *TRF1* overexpression by AAV9 failed to alter telomere length significantly with respect to those observed following treatment with control AAV9 lacking the *TRF1* gene expression cassette. This finding is intriguing, as it means that the authors successfully addressed XPF nuclease-mediated telomere shortening (observed in constitutively *TRF1*-overexpressing TG mice [16]), yet lengthy induction of a telomere-associated gene failed to promote telomere elongation. This is a concern if the system is to be applied to combat age-associated illnesses, where telomere attrition and excessive senescence are the critical therapeutic targets.

Collectively, the available literature on telomerase gene therapy for anti-aging applications properly highlights the promising future and provides exciting evidence to support that transient upregulation of essential telomere maintenance genes does not necessarily translate into carcinogenesis. However, the limited number of available studies means that more extensive exploration of this subject is necessary in the future to clarify what happens when these genes are transiently upregulated in younger mice (less than 1 year of age) and if other gene vectors, either nonintegrative or integrative, could achieve similar results without increasing the risk of carcinogenesis. Despite the potential risks of telomere gene therapy, which hold true for any newly emerging technology, the available results suggest that it is a promising candidate with substantial benefits to combat age-associated illnesses in an aging society with increasing average life expectancy.

### 2.2. Regenerative Application for Cardiovascular Illnesses

In clinical studies, short leukocyte telomere length has been associated with cardiovascular risk factors, such as smoking, obesity, and hypertension [17]: This is likely due to increased oxidative stress, which is frequently observed in various cardiovascular illnesses, causing DNA damage and telomere shortening [18]. Furthermore, low telomerase activity and short telomeres in atherosclerotic plaque cause plaque instability, thus promoting stroke or myocardial infarction (MI). Induction of telomerase expression, albeit at a low level, from the cardiomyocytes of mice following cardiac injury further supports telomerase regulation as being critical for cardiac tissue repair and regeneration [19]. There are numerous reports in which the overexpression of telomerase-related genes can improve growth and inhibit apoptosis of cardiomyocytes, smooth muscle cells, and endothelial cells, whereas inhibition of these genes diminishes cell proliferation [20]. Alternatively, ex vivo-expanded progenitor or stem cells, which can be transplanted in injured tissues to promote neovascularization, can be transduced by telomerase-related genes to enhance their migratory and proliferative capacities [21], thus augmenting the potential therapeutic benefits of these cell therapeutics for treatment of cardiovascular illnesses.

A telomerase-deficient TG mouse with telomere attrition developed cardiomyopathy and exhibited impaired cell division, greater cardiomyocyte death, and cellular hypertrophy, leading to various cardiac dysfunctions [22]. Based on these premises, Bär et al. hypothesized that systemic delivery of cardiotropic AAV9-expressing murine TERT would effectively confer cardioprotective effects in old mice following acute MI [23]. Their findings showed that AAV9 could more effectively transduce cells of heart origin than those from the liver or brain (>60% versus <1.2%), leading to the greatest level of TERT mRNA expression in the heart among various organs following systemic delivery of the vector. Unlike constitutive TERT overexpression by transgenesis leading to cardiac hypertrophy [24], AAV9-mediated TERT transfer did not produce any signs of hypertrophy at 9 to 10 weeks post treatment. Cardiac MI was induced 2 to 3 weeks after AAV administration (due to cardiac transgene expression of AAV9 reaching its peak at 2 weeks post injection and stabilizing after that time [25]). Importantly, TERT induction by AAV9 improved mouse survival and cardiac function post infarction: The infarct size was smaller, interstitial fibrosis was attenuated, and cardiac metabolic activity was rescued, while a stronger repair response and increased proliferation of fibroblasts in scarring tissues were observed. These findings illustrated that nonintegrative telomerase gene transfer can circumvent the cardiac hypertrophy associated with constitutive TERT activation while improving recovery and survival from cardiac infarction in aging mice.

A recent clinical study using autologous cell transplantation of peripheral blood-derived CD34+ cells in 43 patients with cardiomyopathy highlighted that higher expression levels of TERT in cell therapeutics improved their therapeutic effect against cardiovascular illnesses [26], suggesting that potential TERT gene transfer by vectors is a promising strategy to enhance the efficacy of cell therapeutics. These findings illustrated that higher human TERT expression levels within CD34+ cells were associated with better clinical outcomes for patients suffering from cardiomyopathy following cell engraftment by transendocardial delivery. Interestingly, telomere length did not influence the clinical outcomes of cell transplantation therapy; there is a striking similarity with a case covered in Section 2.1, where induction of *TRF1* overexpression, rather than elongation of telomeres, was associated with the longevity of mice [15]. Additionally, these observations are in line with findings by others where ex vivo-expanded endothelial progenitor cells reprogrammed to overexpress human TERT exhibited significantly improved neovascularization and cell survival capacities following cell transplantation in vivo [21]. Although these findings are preliminary at best, they suggest that genetic reprogramming of cardiovascular illness-targeted cell therapeutics for induction of telomerase gene overexpression could be a promising strategy to develop efficient cell/gene fusion therapies in the future.

Despite the presented cases providing strong evidence of potential applications of telomerase gene therapy for cardiovascular illnesses, there are some concerns that require careful consideration during future studies. A study using endothelial cells isolated from patients suffering from coronary artery disease (CAD) demonstrated that cellular senescence in these patient samples was independent of telomere length and was instead dependent on oxidative damage [27], suggesting that senescence was likely induced by stress rather than excessive cellular replication or telomere attrition. Further, a longitudinal study with a large sample size (*n* = 2509) demonstrated that telomeres were not extensively shortened in individuals with familial history of cardiovascular illnesses [28], thus casting a doubt on the telomere hypothesis where telomere attribution contributes to cardiac dysfunction. Additionally, overexpression of TERT in endothelial cells obtained from patients suffering from CAD could not circumvent the cellular senescence caused by biological factors, such as oxidative stress and DNA damage [29], suggesting that telomere attrition caused by aging may not be the key inducer of age-related cardiovascular illness. Although numerous studies have demonstrated a strong association between the shortening of leukocyte telomere length with age and various cardiac malignancies [18], an association does not necessarily establish causality. As the TERT gene therapy case reviewed in this section also targeted adult mice (1 year of age) [23], future studies should approach telomerase-based cardiac gene therapy with caution and carefully consider the optimal therapeutic window for different therapeutics in cardiovascular illnesses with substantial variations in pathophysiology.

### 2.3. Regenerative Application for Anti-Fibrotic Illnesses

Extensive accumulation of damages in the tissues will cause severe shortening of telomeres during the regenerative processes to promote proliferation and differentiation of cells to replace senescent cells with functional copies. Importantly, the chronic damaging of various tissues eventually promotes improper tissue regeneration, resulting in aberrant scarring and fibrosis.

Liver cirrhosis arises due to chronic damage and subsequent conversion of hepatic stellate cells into activated myofibroblast-like cells [30,31]. Sustained hepatocyte turnover following chronic exposure to hepatic damage accelerates telomere attrition and cellular senescence in cirrhotic livers [32,33]. In this regard, Rudolph et al. reported that adenoviral delivery of murine TERC restored telomerase activity and telomere function in a TERC^−/−^ TG mouse model, ultimately resulting in improved liver function and alleviation of cirrhotic pathology [34]. Their findings showed that TERC deficiency-mediated telomere attrition worsened various symptoms (increased induction of apoptosis and decreased hepatocyte proliferation, impaired mitotic progression of regenerating hepatocytes, and extensive cellular fibrosis) of liver cirrhosis following ablation of the liver. To combat these hepatic dysfunctions following hepatic injury in TERC^−/−^ mice with aberrantly short telomere lengths, TERC-expressing adenovirus was systemically administered via the tail vein before liver ablation. TERC-expressing adenovirus effectively transduced the liver tissues, inducing robust transgene expression (due to the well-known hepatic tropism of systemically administered adenovirus [35,36,37,38]). Ad transduction also improved hepatocyte proliferation in TERC^−/−^ mice to a level comparable to that of TERC^+/+^ mice, showing that hepatic damage- and telomere attrition-mediated worsening of cirrhosis can be reversed by TERC gene transfer. Similar hepatic regenerative effects were achieved by others with transposon- and transposase-based integration of the TERT gene into hepatocytes, which enhanced cell growth and conferred protection against chemotoxin CCl_4_ [39], suggesting that several gene transfer vectors, each expressing one of the telomere maintenance-related genes, could be promising candidates to combat liver cirrhosis.

Idiopathic pulmonary fibrosis (IPF) is a rare disease that scars the lungs; the symptoms progressively worsen over time. Similar to liver cirrhosis, telomere shortening following continued wear and tear of the lungs leads to permanent damage accumulation and uncontrolled scarring. Despite few Food and Drug Administration (FDA)-approved drugs currently available for IPF, these drugs only slow disease progression, but are not curative [40,41]. To this end, Povedano et al. demonstrated that pulmonary fibrosis can be combatted by AAV9-mediated transfer of the TERT gene [42]. The group established a progressive lung fibrosis model in TERT^−/−^ TG mice by treating them with low doses of bleomycin (0.5 mg/kg) to closely recapitulate the molecular pathogenesis of clinical IPF, involving both continuous damage and telomere shortening [43]. Their findings illustrate that systemically administered AAV9 effectively transduced 17% of alveolar type II (ATII) lung cells, which are a key cell type for telomere shortening-attributed pulmonary fibrosis. Furthermore, ATII cells accounted for >80% of the AAV9-transduced total lung cell population, suggesting that the vector may have tropism toward ATII cells in lung tissues. Importantly, half of the mice receiving TERT-expressing AAV9 showed undetectable lung fibrosis at 8 weeks post systemic viral administration, whereas all mice treated with an empty AAV9 vector showed severe fibrotic lesions. This was due to AAV9-induced TERT expression lowering collagen accumulation and preventing inflammation (as illustrated by the decreased expression of inflammatory cytokines and lower macrophage infiltration) in the bleomycine-induced IPF TG mouse model. Furthermore, TERT gene transfer prevented senescence and apoptosis in the lungs, as the frequencies of DNA damage response and senescence markers (γH2AX foci, p21, p53, and caspase-3) were markedly lower in these tissues than in those treated with the empty vector. Additionally, their findings illustrate that AAV9-mediated TERT expression downregulated various fibroblast growth factor-related factors in Wnt/transforming growth factor (TGF)-β pathways, whereas regeneration-related genes (DNA replication, DNA repair, and leukocyte transendothelial migration) were upregulated, showing that TERT gene transfer can simultaneously prevent fibrosis and improve the regenerative capacity of the lungs to effectively treat IPF.

### 2.4. Other Potential Applications of Telomerase Gene Transfer

Telomere attrition is a major contributor to several other age-related diseases. This section of the review will discuss how telomere gene therapy is being applied to treat these illnesses.

Genetic mutations to *TERC*, *DKC1* (responsible for production of dyskerin that maintains telomeres), or poly(A) ribonuclease (*PARN*) can lead to reductions in TERC expression levels, and dysregulation of these genes are often observed in patients suffering from severe forms of dyskeratosis congenita (DC) or IPF [44,45,46,47]. Shukla et al. demonstrated that TERC downregulation, which is a major cause of DC, can be overcome by inhibition of the TERC decay pathway with small interfering RNA (siRNA) [44]. Their findings illustrate that siRNA-mediated knockdown of DCP2 and XRN1, which are two components of a cytoplasmic decapping and the 5′–3′ decay pathway, rescued TERC levels in TERC-downregulated cells to 17~28% of the wild-type level. A similar recovery of TERC expression in DKC1 knockdown cells was observed by siRNA-mediated silencing of the nuclear 3′–5′ exonuclease, EXOSC10. Notably, siRNA-mediated knockout of DCP2/XRN1 in combination with EXOSC10 restored TERC expression to ~70% of the wild-type level, suggesting that the silencing of multiple genes involved in the RNA decay pathway can conjointly combat TERC deficiency. Additionally, a reduction in cellular TERC expression levels caused by pathogenic point mutations to TERC subdomains, which are observed in DC patients, was also effectively treated by siRNA-mediated knockdown of DCP2, XRN1, or EXOSC10. The authors illustrated that a deficiency of functional PARN, which normally contributes to TERC stability by removing oligo(A) tails, lowers TERC expression, and that this effect can be partially reversed by siRNA-mediated knockdown of EXOSC10, suggesting that siRNA-mediated systematic knockdown of RNA destabilizing factors can combat a TERC deficiency caused by pathological dysfunction of PARN, TERC, or DKC1.

Degenerative disc disease (DDD) is characterized by progressive loss of human nucleus pulposus (HNP) cells and extracellular matrix (ECM) [48]. As HNP cells promote massive deposition of ECM, cell therapy supplementing HNP cells to degenerative discs has been actively investigated. One of the major hurdles to this approach is the limited proliferation capacity and progressive loss of the ECM synthesis ability of HNP cells during in vitro expansion [49], which complicates the acquisition and security of sufficient HNP cells. To this end, AAV vector-mediated transduction of the TERT gene to nucleus pulposus cells has been shown to improve proliferation and in vitro maintenance of nucleus pulposus cells [50,51]. One of the shortcomings of this approach is that TERT expression by replication-deficient AAV, which predominantly exists in cells as an episomal concatemer outside the chromosome [52], was eradicated within 150 days of transduction. Wu et al. proposed that integration of the TERT gene via the lentiviral vector (LV-TERT) may overcome AAV-mediated finite TERT expression in HNP cells [53]. Their findings showed that LV-TERT effectively infected freshly isolated HNP cells, as 84% of cells were transduced by 7 days post transduction. Unexpectedly, LV-TERT-mediated TERT expression also progressively decayed over time, and TERT expression was only observed for up to 150 days after the initial transduction (complete absence by day 210). Still, LV-TERT-treated HNP cells showed greater telomere lengths up to 210 days post transduction compared to mock-transduced HNP cells, as well as a significantly shortened doubling time of these cells from passage 8 onward. These LV-TERT-infected HNP cells also retained their collagen II and aggrecan secreting ability at a higher level than mock-transduced cells up to 150 days post viral transduction. These studies with either AAV or lentiviral vectors for the transfer of TERT into HNP cells suggest that the shortage of transplantable cells for treatment of DDD could be addressed by gene therapy.

Aplastic anemia is a potentially life-threatening heterogeneous disorder, where bone marrow (BM) cannot produce sufficient quantities of new blood cells due to a sharp decline in the quantity of immature hematopoietic stem and progenitor cells (HSPC) [54]. The disease can occur either in a hereditary or acquired manner. A subpopulation of either inherited or acquired forms has been identified with replicative impairment of HSPC due to telomere attrition. To address these problems of aplastic anemia associated with telomere shortening, Bar et al. utilized TERT-expressing AAV9 in two different aplastic anemia-suffering TG mouse models (partial deletion of the *TRF1* gene in BM by the CreLox system or constitutive silencing of TERT in TERT^−/−^ to induce telomere shortening) [55]. Upon systemic administration of AAV9, 2% of all BM cells were positive for the AAV9 transgene, and TERT expression was maintained up to 8 months after the initial treatment. Either a 10- or a 3.5-fold increase in TERT expression was observed in the blood-forming compartments of the BM (HSPCs or lineage-committed BM cells, respectively) following administration of TERT-expressing AAV9 compared to those treated with an empty vector. Importantly, selective and partial deletion of *TRF1* in the BM of TG mice led to rapid telomere shortening, replicative senescence, BM failure, and a poor survival rate (55% at 100 days post viral injection) in the absence of any TERT gene transfer, whereas mice receiving TERT-expressing AAV9 showed a greatly improved survival rate (87%) due to the lower incidence rate of aplastic anemia (empty versus TERT vectors: 44% and 13%, respectively). Impressively, TERT gene transfer led to a drastic improvement in telomere length maintenance, as the length was retained at a level similar to that prior to deletion of *TRF1* by the CreLox system, whereas the group treated with the empty AAV9 vector exhibited a 12-kb decrease in telomere length. This is important, as the degree of telomere shortening was positively correlated with the incidence rate of aplastic anemia. Similar anti-anemic effects were observed following TERT gene transfer by AAV9 in the TERT^−/−^ model; this was evidenced by higher erythrocyte and platelet counts in the gene transfer group compared to the empty vector-treated group. Collectively, these findings showed that telomere attrition, which causes BM dysfunction and aplastic anemia in patients, can be effectively treated by TERT gene transfer.

## 3. Anticancer Application

In most cases, cancer is an age-related genetic disease that occurs with persistent accumulation of genomic instability over time [3]. Cancer cells frequently acquire mechanisms to bypass the senescence that naturally occurs with telomere attrition and to achieve immortality. The most frequently adopted strategy for telomere maintenance by cancer cells is reactivation of TERT, which is upregulated in ~85% of clinical cases. The high prevalence of TERT reactivation in cancer patients and its integral role in cancer immortality indicate TERT as an attractive therapeutic target for cancer therapy. Thus, this section of the review will discuss how the constitutively active TERT pathway is being targeted or utilized to enhance the therapeutic efficacy or safety profile of various gene therapeutics.

### 3.1. Endowing Cancer Specificity to Gene Therapeutics by the TERT Promoter

The immortality of cancer is maintained by constitutive TERT expression, which means that the intracellular environment is rich in various factors that activate TERT promoters, while the promoter remains largely inactive in most normal cells. This allows TERT promoter-driven gene expression to occur in a cancer-specific manner in the majority of cancers, as ~85% of all cancers exhibit overexpression of TERT. This pan-cancer applicability of the human TERT promoter is important when considering that most other cancer-specific promoters are only capable of selectively targeting a small subset of cancers in a tissue-specific manner. For example, human alpha fetoprotein, carcinoembryonic antigen, and MUC1 promoter can only target subsets of hepatocellular carcinoma, colorectal and lung cancers, or breast cancer, respectively [56]. The most common adaptation of TERT promoter-driven gene therapy simply expresses various therapeutic moieties (anticancer transgenes, miRNA, or the CRISPR/Cas9 system) in a cancer-selective manner [57,58,59,60,61,62].

Many of the pioneering works (from the early 21st century) utilizing human TERT promoter focused on adenovirus-mediated delivery of suicide genes [63,64] or apoptosis-inducing genes [65,66,67] that are capable of directly inducing cancer cell death. Replication-incompetent adenoviruses were chosen as a vector of choice in these studies due to their well-characterized and highly efficacious gene transfer property. All therapeutic transgene expression (for both proapoptotic genes and prodrug converting catalytic enzymes) by these systems occurred in a cancer-specific manner and at a high level in a wide range of cancer types of both human and murine backgrounds (fibrosarcoma, lung adenocarcinoma, colon cancer, osteosarcoma, etc.), demonstrating the broad applicability of human TERT promoter-driven gene therapeutics. In marked contrast, minimal transgene expression was observed in normal somatic cells and BM progenitor cells, demonstrating minimal acute and long-term toxicity. These works illustrated that human TERT promoter-driven gene therapeutics can target a wide range of cancers and express therapeutic genes in a safe manner.

During the past decade, technological breakthroughs have increased the practicality of genome editing. The most recent adaptation to the field is CRISPR/Cas9, which is a simple RNA-guided platform with highly efficient and specific genome editing in diverse organisms [68]. Thus, the CRISPR/Cas9 system has emerged as a revolutionary tool for biomedical research, facilitating new possibilities for treating various diseases [69]. In 2017, the first report of a human TERT promoter-driven GAL4/upstream activating sequence (UAS) binding system for selective activation of CRISPR/Cas9 targeting the HRAS oncogene in bladder cancer cells was published [62]. Specifically, human TERT promoter drives GAL4 expression in a cancer-specific manner, and GAL4 subsequently binds to UAS to activate a promoter driving Cas9 nuclease expression, ultimately promoting preferential Cas9 expression in cancer cells through a genetic cascade. A lentivirus expressing an HRAS-targeting guide RNA, GAL4, and UAS-activated Cas9 nuclease (HRAS-LV) induced the silencing of HRAS in bladder cancer cell lines (<50% expression level observed following treatment with negative control lentivirus (NC-LV)), whereas the HRAS was unaffected in human foreskin fibroblasts, illustrating the cancer-specific silencing of HRAS by the human TERT promoter-driven CRISPR/Cas9 genetic circuit. Selective silencing of HRAS by HRAS-LV also led to selective inhibition of bladder cancer cell proliferation, migration, and invasion, while increasing apoptotic cancer cell death compared to NC-LV-treated groups, showing that a TERT promoter-driven and CRISPR/Cas9-based genetic circuit can be a promising strategy to selectively silence oncogenes of cancer.

#### TERT Promoter-Driven Replication of Oncolytic Adenoviruses

Similar to the application discussed in Section 3.1, the human TERT promoter can enable cancer-specific replication of adenoviruses by controlling the key replicative genes (such as viral E1A gene), ultimately generating oncolytic adenoviruses [70,71,72]. The pioneering works in human TERT promoter-driven oncolytic adenoviruses were published in 2003, when three separate reports demonstrated that human TERT promoter-driven expression of the adenoviral E1A gene endowed cancer specificity on adenoviral replication [73,74,75]. Two out of the three reports utilized unmodified human TERT promoters to drive the adenoviral E1A expression and showed that these oncolytic adenoviruses induced tumor growth suppression up to 9 or 30 days post treatment ([75] or [74], respectively).

Despite numerous reports showing that human TERT promoter can endow cancer specificity to gene therapeutics, the promoter induces relatively weak transgene expression. This shortcoming remains a major challenge toward achieving an optimal therapeutic index with a TERT promoter-based gene expression system. To this end, Kim et al. developed a modified human TERT (mTERT) promoter to drive the replication of oncolytic adenovirus and compared its antitumor efficacy with that of unmodified human TERT promoter-driven oncolytic adenovirus [73]. Their findings demonstrated that the mTERT promoter (containing additional Sp1 and c-Myc binding sites) induces a higher level of transgene expression than unmodified human TERT promoter in various TERT-positive cancer cells (up to 10-fold higher gene expression), showing that promoter activity can be improved in cancer through insertion of additional binding sites for oncogenic transcription factors upstream of the promoter. Further, mTERT promoter-driven replication of oncolytic adenovirus improved the potency and duration of antitumor efficacy compared to those achieved by control oncolytic adenovirus replicating under the control of unmodified human TERT promoter. Notably, mTERT promoter-driven oncolytic adenovirus induced complete regression in 40% of mice by 10 days after virus injection, and no palpable tumor was observed for up to 2 months, showing more potent and durable tumor growth inhibition than those achieved by the other human TERT-driven oncolytic adenoviruses reported in 2003. In addition, the mTERT promoter-driven oncolytic adenovirus exhibited markedly higher cancer specificity than did the oncolytic adenovirus replicating under the control of the unmodified human TERT promoter. Together, these findings showed that modification of the TERT promoter was necessary to improve both the cancer specificity and cytolytic effect of the oncolytic adenovirus.

In 2018, two different modification approaches were reported to improve mTERT promoters further. The first approach generated a hybrid cancer-specific promoter by combining E2F and mTERT promoters with several copies of hypoxia response elements (HRE) to generate two variations of hypoxia-responsive and cancer-specific promoters (HEmT or HmTE) [71]; HEmT showed higher transcriptional activity than HmTE and illustrated that the order of individual promoter components greatly affects the promoter activities of hybrid promoters. Systemically administered HEmT promoter-driven oncolytic adenovirus elicited more potent tumor growth inhibition than a clinically approved oncolytic adenovirus (Oncorine) in a highly aggressive orthotopic pancreatic tumor model through more active replication of adenovirus in both normoxic and hypoxic tumor regions. The second approach inserted additional oncogenic transcription factor binding domains [72]. In this report, the mTERT promoter was further improved by incorporating six copies of HRE and five copies of c-Myc binding sites upstream of mTERT (generating H5CmTERT) to drive replication of the oncolytic adenovirus. This H5CmTERT promoter-driven oncolytic adenovirus replicated more efficiently than the mTERT-driven oncolytic adenovirus in cancer cells under both normoxic and hypoxic conditions, ultimately enhancing antitumor efficacy and overcoming the commonly observed downregulation of virus replication in the hypoxic tumor region.

Collectively, these studies illustrated that human TERT promoter can endow cancer specificity to the oncolytic adenovirus through transcriptional regulation of the E1A gene, and that the potency of these vectors can be further improved through genetic modification of the human TERT promoter.

### 3.2. Downregulation of Telomere-Related Genes in Cancer by Gene Therapy

Alternative applications within the scope of gene therapy exploiting constitutive activation of TERT in cancer utilize silencing or inhibition of TERT or TERC activity [76,77,78]. For example, siRNA or antisense RNA targeting TERT has been an actively investigated gene therapeutic of choice [79,80,81,82,83,84], with a small number of reports exploring different strategies (such as expression of c-terminal TERT polypeptide (hTERTC27) [85,86,87], CRISPR/Cas9 targeting an oncogenic TERT promoter mutant allele that enables constitutive activation in cancer [88], or microRNA-mediated downregulation of TERT by targeting of the 3′-UTR region [89]). In general, these approaches promote cellular senescence, induce apoptotic tumor cell death, and attenuate the rate of proliferation.

The earliest works in the scope of the silencing of TERT or TERC were achieved by direct delivery of antisense oligonucleotides or siRNA with transfection reagents [90,91]. A report utilizing an antisense oligonucleotide targeting TERC showed that the antisense oligonucleotide effectively attenuated TERC expression and inhibited TERT activity in TERT-positive cancer cells, leading to cell death through a more robust induction of apoptosis specifically in TERT-positive cancer cells [91]. The viability of TERT-negative cells was unaffected by antisense oligonucleotide treatment. Interestingly, the antisense oligonucleotide treatment did not affect telomere length; thus, the reduction in the viability of TERT-positive cancer cells was not due to telomere attrition. One plausible explanation for this telomere maintenance-independent pro-apoptotic effect mediated by the antisense oligonucleotide is through the non-canonical function of telomerase. In detail, a catalytically active mutant form of TERT without the ability to elongate telomeres has been reported to cause oncogenic transformation [92], suggesting that TERT exerts non-canonical (telomeric-independent) functions. Further, alternatively spliced variants of TERT, which lack telomerase activity, are capable of inducing the expressions of growth-promoting genes and protect cancer cells from apoptosis [93]. This literatures suggests that TERT-targeted antisense oligonucleotide [91], which selectively binds and targets only a portion of TERT in cancer, may exert a pro-apoptotic effect through silencing of the non-canonical function of TERT, thus telomere length is unaffected. Kosciolek et al. evaluated the therapeutic efficacy of siRNA targeting either TERC or TERT [90] and showed that both types of siRNA could effectively attenuate telomerase activity in cancer cells. Their findings showed that siRNA targeting TERC was more effective in inhibiting telomerase activity than was siRNA targeting TERT. This does not conclusively indicate that TERC is a better therapeutic target for siRNA-mediated silencing of telomerase than TERT, as only one siRNA candidate for each target was evaluated in this study. The siRNA-mediated silencing of telomerase activity decreased rapidly with time. In a similar context, siRNA-mediated inactivation of telomerase was more effective when siRNA administration was divided into multiple regimens during the course of treatment rather than a one-time injection of the same siRNA quantity at the beginning of treatment. These results clearly demonstrated the relatively short duration of the siRNA-mediated gene silencing effect and are in line with the literature showing the short biological activity and poor stability of siRNA as major roadblocks slowing the path to clinical translation [94,95].

One promising strategy to circumvent the poor biological stability and short-term silencing effect mediated by RNA interference therapeutics in the nucleotide form is to deliver short hairpin RNA (shRNA) via an expression vector. Unlike siRNA treatment, where 99% of duplex siRNA is degraded within 48 h of cellular delivery, shRNA transcription in the nucleus by an expression vector is continuous and leads to more durable gene silencing than siRNA [96]. In support, the plasmid-based delivery of shRNA targeting either TERT or TERC led to durable suppression of bladder cancer cell growth up to 6 days after treatment [78]. Interestingly, two plasmids expressing the shRNA candidate with the strongest silencing effect toward either TERT or TERC (among three shRNA candidates for each target gene) showed similar cancer cell growth inhibitory effects. These discrepancies in therapeutic effects by RNA interference therapeutics targeting TERC or TERT among several reports show that systematic and more extensive analyses are necessary in the future to determine which of the two genes is the optimal target for RNA interference. The combination of the two plasmids (each targeting either TERT or TERC) led to the strongest bladder cancer growth inhibition, but the underlying mechanism behind this strong combinatory effect was not elucidated in this report and requires further exploration. A similar expression vector-based shRNA approach for the silencing of TERT and TERC was also explored with adenovirus [77]. One potential advantage of using an adenoviral vector over plasmids is its superior gene transfer efficiency through an effective endogenous endocytosis mechanism, which means that viral vectors, unlike plasmid or DNA vectors, can be internalized into cells without the assistance of a carrier [97]. Treatment of oral squamous cell carcinoma (OSCC) cells with adenovirus expressing TERC-targeted shRNA (Ad-shTERC) led to a time-dependent increase in the inhibition of TERC mRNA expression up to 3 days post treatment, and adenovirus expressing TERT-targeted shRNA (Ad-shTERT) similarly inhibited TERT mRNA expression [77]. Both shRNA-expressing adenoviruses suppressed telomerase activity in a dose-dependent manner, with Ad-shTERC showing a superior inhibitory effect compared with Ad-shTERT or the combination of two adenoviruses (Ad-shTERC + Ad-shTERT) administered at the same total viral dose as the monotherapies. Inhibition of telomerase activity also correlated with suppression of OSCC cell proliferation and tumor growth, where the inhibitory effects were ranked as follows: Ad-shTERC > Ad-shTERC + Ad-shTERT > Ad-shTERT. Collectively, these studies comparing the gene silencing and therapeutic effects of the vector-mediated expression of shRNA targeting either TERT or TERC show that more durable silencing of telomerase-related genes can be achieved by shRNA than by siRNA.

Although RNA interference is the most commonly investigated gene therapy strategy to disrupt the telomerase function in cancer, there are several other approaches being investigated. One of these approaches utilizes a vector-mediated expression of hTERTC27 to cause telomere dysfunction [98]. hTERTC27 is a 27-kDa C-terminal human TERT polypeptide lacking several domains, such as conserved reverse transcriptase motifs and TERC-binding domains of wild-type TERT, which are critical for telomerase activity. Unlike RNA interference therapeutics that directly suppress the expression of TERC or TERT to disrupt telomere maintenance and in cancer, hTERTC27 neither suppresses the expression levels of these genes nor restricts telomerase activity [99]. The anticancer activity of hTERC27 in TERT-positive cancer cells occurs through upregulation of the senescence-associated gene, p21 [99], or by promoting cells to undergo senescence-like growth arrest and apoptosis through nuclear localization of hTERTC27 polypeptide, causing chromosome end-to-end fusion [98]. The indirect effect of hTERTC27 could be beneficial for long-term therapeutic applications, since direct inhibition of telomerase activity (as in the case of RNA interference) may exert selective pressure for cancer cells to adapt alternative lengthening of telomeres to maintain telomeres in a telomerase-independent manner [100,101]. Importantly, stable hTERC27 expression in TERT-positive cancer cells via lentivirus attenuated tumorigenicity compared to parental cancer cells. In a separate report, AAV-mediated expression of hTERTC27 was also shown to elicit potent antitumor effects against glioma by more robust induction of apoptosis [85]. These studies showed that vector-mediated expression of hTERTC27 can be a viable strategy to treat cancers.

A C>T single-nucleotide mutation at either the -124 or -146 position (with respect to the ATG site of TERT) of the TERT proximal promoter region is frequently observed in various types of tumors [102]. This mutation has been reported to generate an ETS1 transcription factor binding site, thus leading to greater transcriptional activity of these mutant TERT promoters in comparison to the wild-type TERT promoter. CRISPR/Cas9-induced correction of oncogenic TERT promoter mutation has been shown to attenuate telomerase activity in urothelial cancer cells [88]. To achieve this goal, a donor plasmid containing a wild-type TERT promoter allele was co-transfected with a plasmid co-expressing Cas9 and C124T mutant TERT promoter allele-targeted guide RNA. This approach successfully led to replacement of the mutant TERT allele with a wild-type allele in the SCaBER cancer cell line, which normally contains one wild-type and C124T mutant TERT allele, leading to a 40% to 50% reduction in telomerase activity, impairment of telomere elongation, and inhibition of cell growth compared to the parental cancer cell line. These findings suggest that oncogenic activation of the TERT promoter in cancer can be reversed by CRISPR/Cas9-based replacement of the oncogenic TERT promoter allele with a wild-type allele.

Collectively, these studies demonstrate that gene therapeutics with variable modes of telomerase inhibitory effects can be promising candidates to treat a wide range of tumor types in the future.

## 4. Conclusions

Upregulation and downregulation of telomerase-related genes have shown promising therapeutic results for anti-aging/regenerative and anticancer applications, respectively. Furthermore, the preliminary safety hazard assessments for vector-mediated expression of telomerase-related genes in anti-aging or regenerative applications suggest that nonintegrative expression of these genes does not promote oncogenesis. Importantly, the transfer of telomere maintenance-related genes improved longevity and tissue regeneration to combat aging and age-related diseases. Alternatively, the strategies that silence or disrupt telomerase activity in cancer cells or endow cancer specificity to gene therapeutics via utilization of the human TERT promoter have rapidly evolved since their conception and development in the beginning of this century, ultimately improving the therapeutic potency and safety profile of cancer gene therapy. Collectively, these reports illustrate that telomere-associated genes are attractive candidates for gene therapy with multidimensional therapeutic benefits and wide applicability toward numerous disease targets.
